# Overexpression of Human sFLT1 in the Spongiotrophoblast Is Sufficient to Induce Placental Dysfunction and Fetal Growth Restriction in Transgenic Mice

**DOI:** 10.3390/ijms25042040

**Published:** 2024-02-07

**Authors:** Rebekka Vogtmann, Alina Riedel, Ivanka Sassmannshausen, Sarah Langer, Elisabeth Kühnel-Terjung, Rainer Kimmig, Hubert Schorle, Elke Winterhager, Alexandra Gellhaus

**Affiliations:** 1 Department of Gynecology and Obstetrics, University Hospital, 45147 Essen, Germanyalina.riedel@uk-essen.de (A.R.); rainer.kimmig@uk-essen.de (R.K.); 2 Department of Developmental Pathology, Institute of Pathology, University Hospital Bonn, 53127 Bonn, Germany; schorle@uni-bonn.de; 3 EM Unit, Imaging Center Essen, University Hospital, 45147 Essen, Germany; elke.winterhager@uk-essen.de

**Keywords:** sFLT1, preeclampsia, fetal growth restriction, pregnancy, spongiotrophoblast, placenta, transgenic mouse model, angiogenesis

## Abstract

Preeclampsia (PE) is characterized by maternal hypertension and placental dysfunction, often leading to fetal growth restriction (FGR). It is associated with an overexpression of the anti-angiogenic sFLT1 protein, which originates from the placenta and serves as a clinical biomarker to predict PE. To analyze the impact of sFLT1 on placental function and fetal growth, we generated transgenic mice with placenta-specific human sFLT1 (hsFLT1) overexpression. Immunohistochemical, morphometrical, and molecular analyses of the placentas on 14.5 dpc and 18.5 dpc were performed with a focus on angiogenesis, nutrient transport, and inflammation. Additionally, fetal development upon placental hsFLT1 overexpression was investigated. Dams exhibited a mild increase in serum hsFLT1 levels upon placental hsFLT1 expression and revealed growth restriction of the fetuses in a sex-specific manner. Male FGR fetuses expressed higher amounts of placental *hsFLT1* mRNA compared to females. FGR placentas displayed an altered morphology, hallmarked by an increase in the spongiotrophoblast layer and changes in labyrinthine vascularization. Further, FGR placentas showed a significant reduction in placental glycogen storage and nutrient transporter expression. Moreover, signs of hypoxia and inflammation were observed in FGR placentas. The transgenic spongiotrophoblast-specific hsFLT1 mouse line demonstrates that low hsFLT1 serum levels are sufficient to induce significant alterations in fetal and placental development in a sex-specific manner.

## 1. Introduction

In human preeclampsia (PE), the placenta is considered to be the origin of the elevated serum levels of anti-angiogenic soluble fms-like tyrosine kinase-1 (sFLT1) protein. Cerdeira et al. 2019 [1] demonstrated that pregnant women with PE exhibit a significant sFLT1 gradient between the uterine and peripheral veins, with the highest sFLT1 levels in the placenta and lowest in the periphery. As one of the most common pregnancy disorders in the world, PE affects 3–5% of all pregnancies and remains a leading cause of mortality and morbidity of the fetus and mother [2]. PE is characterized by placental dysfunction and abnormal perfusion which is caused by inadequate spiral artery (SpA) remodeling, often resulting in fetal growth restriction (FGR) [3]. The adverse fetal and placental development can lead to long-term consequences and an increased risk of chronic illnesses such as cardiovascular diseases or metabolic disturbances like diabetes in the offspring [4].

There are many studies about preeclampsia and the changes in placental morphology and function, mostly revealed by investigating rodent models [5,6,7,8,9], but only few studies exist regarding the exact role of sFLT1 in these processes. It is well known that trophoblast cells are the origin of sFLT1 that enters the maternal circulation during pregnancy, leading to the hypothesis that abnormal trophoblast formation and therefore excess sFLT1 is the primary cause of maternal vascular dysfunction in this disease through its sequestration of VEGF [10]. Abnormal trophoblast invasion into the maternal decidua has been intensely studied in PE [11]. A recent single-cell transcriptomic study in human pregnancy revealed a cell-autonomous dysregulation of FLT1 and PLGF transcription in the syncytial trophoblast in early but not late preeclampsia [12]. This study also confirmed that PLGF was mainly derived from the syncytiotrophoblast and was significantly downregulated in early-onset PE, whereas FLT1 expression was upregulated across all trophoblast cell types in the human PE placenta. As reviewed by Yagel et al. (2023) [13], type I early-onset preeclampsia is characterized by placental dysfunction or malperfusion, shallow trophoblast invasion, inadequate SpA remodeling, profound syncytiotrophoblast stress, elevated sFLT1 levels, reduced PLGF levels, and high peripheral vascular resistance, often leading to FGR. Type II preeclampsia typically occurs in the later stages of pregnancy with a moderately dysfunctional placenta and a normal or slightly disturbed sFLT1/PlGF ratio.

Since the exact role of overexpressed placental sFLT1 in PE on placental as well as on fetal development has been inadequately investigated, we established a transgenic human sFLT1 (hsFLT1) placenta-specific mouse model to study this. In previous studies [8], we used an existing PE mouse model published by Kumasawa et al., 2011, [5] that uses lentivirus-induced placental overexpression of hsFLT1. As a result, the placenta-derived hsFLT1 led to FGR, a significantly lower placental weight, and decreased placental size due to a reduced labyrinthine layer [8]. However, this mouse model suffered from several disadvantages such as a high variability in outcome as each animal had to be generated individually. Moreover, the transduction of the blastocyst means that hsFLT1 is already expressed before implantation, which differs from the human disease pattern and most likely has an impact on placental development. Recently, we established a transgenic hsFLT1/rtTA mouse model for PE and FGR with systemic, not placenta-specific, induction of hsFLT1 by doxycycline [14,15,16]. To overcome the challenges and to mimic the human PE pathology more closely, we developed here a transgenic hsFLT1/tTA/Tpbpa-Cre mouse model to achieve stable placenta-specific hsFLT1 overexpression via three different transgenic alleles that are independent from external induction factors. By design, hsFLT1 overexpression is induced exclusively in the spongiotrophoblast layer through Tpbpa-specific expression of Cre recombinase. Because of this Tpbpa-Cre-driven gene expression, hsFLT1 expression starts at around 8.5 days post conception (dpc) which corresponds to the second trimester of murine pregnancy, similar to the sFLT1 expression pattern in human pregnancy. By using this transgenic mouse model, we had the chance to investigate the pathophysiological mechanisms occurring upon placenta-specific human sFLT1 overexpression leading to an impairment of placental and fetal development, as is seen in human PE.

## 2. Results

### 2.1. Human Soluble Fms-like Tyrosine Kinase Receptor-1 (hsFLT1) Is Exclusively Expressed in Spongiotrophoblast Cells in Transgenic Mice

**Figure 1:**
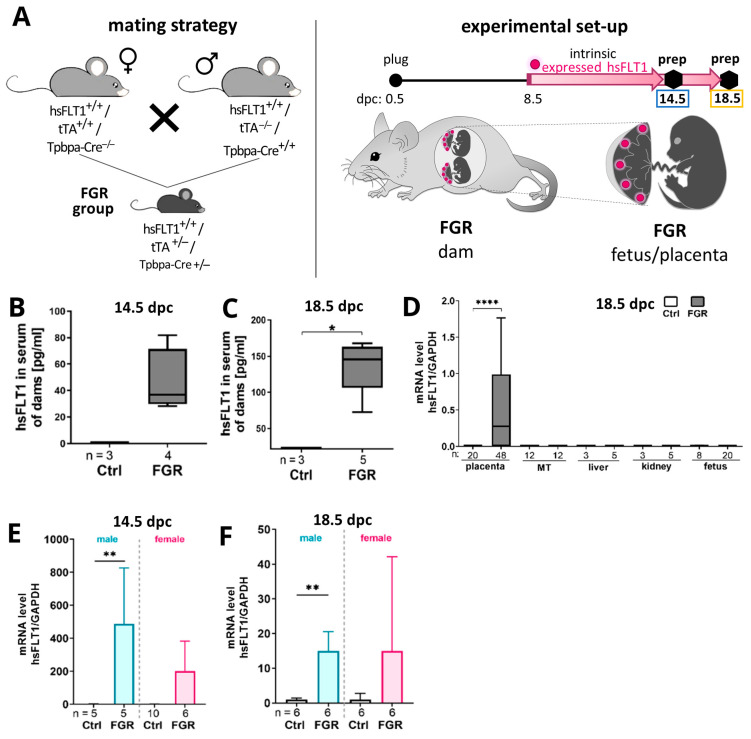
Generation of hsFLT1/tTA/Tpbpa-Cre mouse model to induce placenta-specific hsFLT1 overexpression. (**A**) Mating scheme (**left**) and experimental setup (**right**) to generate hsFLT1-expressing placentas in filial generation 1 of the FGR group. At 8.5 dpc, the placenta-specific hsFLT1 overexpression intrinsically starts and is limited to the spongiotrophoblast cells of the placenta (red dots). Preparations were either performed at 14.5 dpc or at 18.5 dpc. (**B**,**C**) hsFLT1 in serum was exclusively present in dams of the FGR group at 14.5 (**B**) and 18.5 dpc (**C**). (**D**) *hsFLT1* mRNA expression was only detected in placentas of FGR fetuses and not in the mesometrial triangle (MT) or other maternal (liver, kidney) or fetal tissues (**E**,**F**). Placental *hsFLT1* mRNA expression was significantly higher in the male FGR group compared to the male Ctrl group at 14.5 dpc (**E**) and 18.5 dpc (**F**). The expression level of *hsFLT1* was determined by qPCR and normalized to the housekeeping gene *Gapdh*. Data are presented in a box plot with median, interquartile range, and lower/upper extremes or in a bar graph with standard error. n = number of dams (**B**–**C**), maternal/fetal tissue (**D**), or placentas (**D**–**F**). * *p* ≤ 0.05, ** *p* ≤ 0.01, and **** *p* ≤ 0.0001 as determined by Mann–Whitney test.

The functionality of the hsFLT1/tTA/Tpbpa-Cre mouse model with intrinsic placenta-specific hsFLT1 overexpression starting at 8.5 dpc (Figure 1A) was verified by measuring the serum hsFLT1 level in pregnant dams at 14.5 and 18.5 dpc (Figure 1B,C). Serum levels of hsFLT1 in dams were only detected in dams with placenta-derived hsFLT1 overexpression and not in the Ctrl group. At midgestation (14.5 dpc), the average concentration was 36.85 pg/mL and ranged from 33.76 to 81.97 pg/mL. At term (18.5 dpc), the serum hsFLT1 level increased to an average of 136.7 g/mL, with a range of 72.31 to 167.9 pg/mL. To validate the placenta-specific expression, hsFLT1 mRNA levels were measured at 18.5 dpc in the placenta as well as in dam tissues such as the mesometrial triangle (MT), liver, and kidney and in fetal tissue. hsFLT1 could only be detected in placentas from FGR samples but not in other maternal or fetal organs (Figure 1D). At both time points, placentas of male fetuses with FGR displayed higher *hsFLT1* transcript levels compared to placentas of female fetuses (Figure 1E,F).

### 2.2. Placenta-Specific hsFLT1 Overexpression Resulted in Growth-Restricted Fetuses

**Figure 2:**
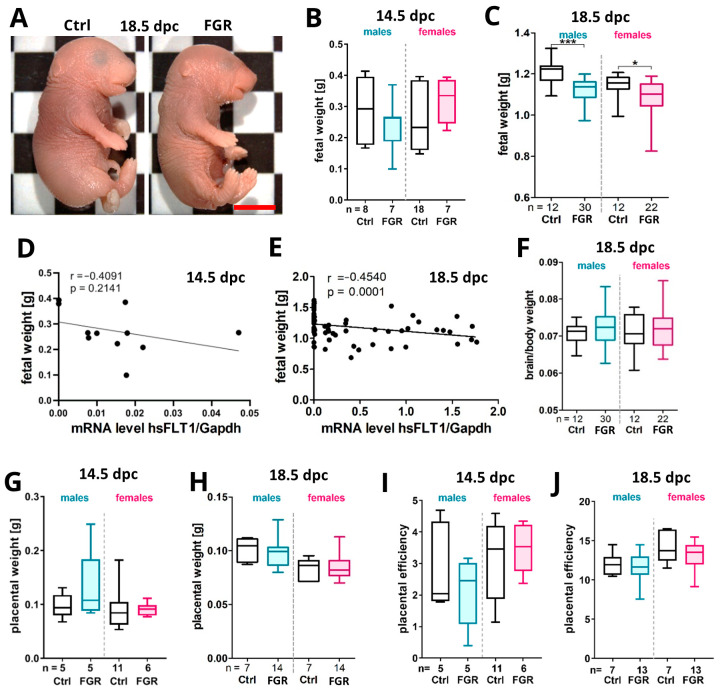
Impact of placenta-specific hsFLT1 overexpression on fetal and placental weight. (**A**) Representative fetal phenotypes for Ctrl and FGR at 18.5 dpc. (**B**) Fetal weight was decreased in the male FGR group and increased in the female FGR group at 14.5 dpc. (**C**) Fetal weigh was significantly decreased in both male and female FGR groups at 18.5 dpc compared to controls. (**D**,**E**) Fetal weight did not correlate to the hsFLT1 mRNA level at 14.5 dpc (**D**) but strongly correlate at 18.5 dpc (**E**). (**F**) Brain/body weight ratio did not show a difference between the experimental groups. (**G**,**H**) Placental weight was slightly increased in the male and female FGR groups at 14.5 dpc (**G**), whereas it was decreased at 18.5 dpc compared to the respective Ctrls (**H**). (**I**,**J**) The placental efficiency was not affected at 14.5 (**I**) and 18.5 dpc (**J**) upon placenta-specific hsFLT1 overexpression. Data are presented in a box plot with median, interquartile range, and lower/upper extremes. n = number of fetuses or placentas. * *p* ≤ 0.05, and *** *p* ≤ 0.001 as determined by the Mann–Whitney test. Statistical analyses of correlation were calculated by Spearman r test. Scale bar: 1 mm.

To detect the impact of placenta-specific hsFLT1 expression on placental and fetal development, the litter size, number of resorptions, sex ratio, fetal body and organ weight, as well as placental weight were recorded (Figure 2 and Figure S1). At 14.5 dpc, the litter size was smaller and resorptions were only present in the FGR group (Figure S1B,C). At 18.5 dpc, the influence of placental sFLT1 on litter size and resorptions was the opposite compared to the effects at 14.5 dpc (Figure S1E,F). These results may indicate an earlier loss of fetuses in the experimental group compared to the controls which approximate the litter size at term between both groups. The sex distribution did not differ significantly in the FGR group at both time points (Figure S1D,G), and was nearly balanced.

The fetuses of the FGR group appeared slimmer at 18.5 dpc compared to the controls (Figure 2A). The fetal weight was reduced but with high variations in the male FGR group at 14.5 dpc, whereas the female FGR group showed an increased fetal weight compared to its respective Ctrl at this time point (Figure 2B). At term, this weight reduction tendency in males increased to a significant difference, similar to the changes seen in female weight but to a lesser degree (Figure 2C). The fetal weight was significantly correlated with the transcript level of hsFLT1 at 18.5 dpc, but not at 14.5 dpc (Figure 2D,E). The brain/body weight, the placental weight, as well as the placental efficiency (fetal weight/placental weight) was not affected in hsFLT1 fetuses (Figure 2F–J). But, interestingly, at 18.5 dpc, the brain weight was significantly reduced and the liver/body weight was significantly increased only in the male FGR group compared to the Ctrls (Figure S1H,M), whereas the kidney weight and the kidney/body weight was significantly enhanced in the FGR group, but only in females (Figure S1K,O). No changes in the liver and heart weights as well as in the brain/liver or heart/body ratio were observed in both groups (Figure S1I,J,L,N).

### 2.3. Expression of Placental hsFLT1 Caused an Increase in Size of the Placental Spongiotrophoblast

**Figure 3:**
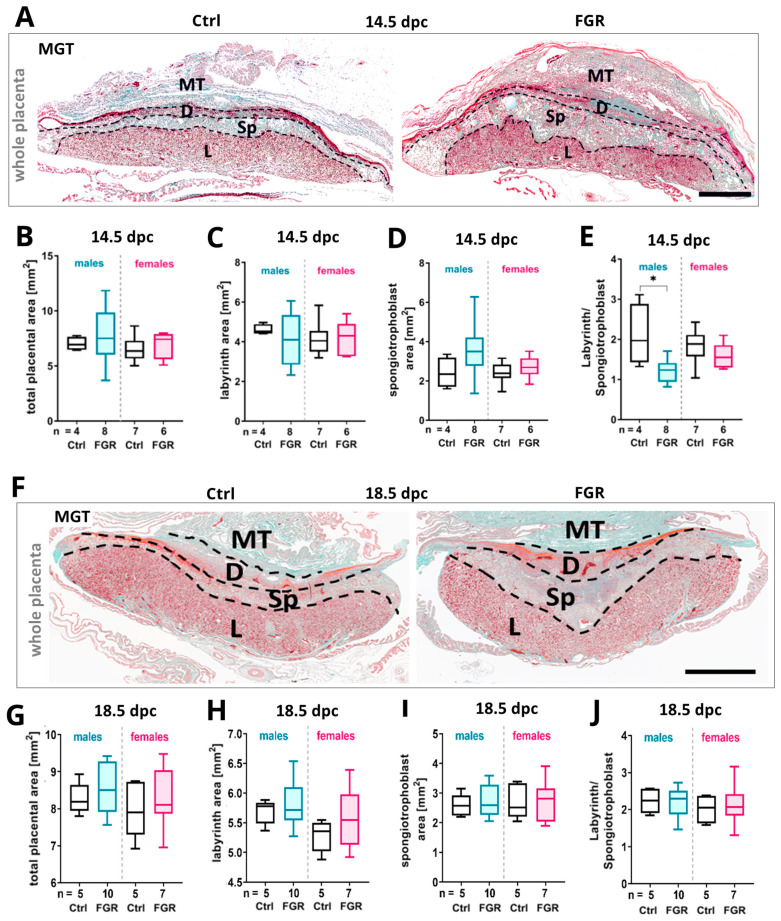
Morphometric analysis of placentas upon placenta-specific hsFLT1 overexpression. (**A**) Overview of placental compartments in MGT-stained placental sections of Ctrl and FGR groups at 14.5 dpc. (**B**–**D**) Total placental area (**B**), labyrinth area (**C**), and spongiotrophoblast area (**D**) were slightly increased in both FGR groups at 14.5 dpc; only the labyrinth area in male FGR placentas was decreased. (**E**) The labyrinth/spongiotrophoblast ratio was significantly decreased in the male FGR group compared to the male Ctrl group at 14.5 dpc. (**F**) Representative MGT-stained placental sections of control and FGR groups at 18.5 dpc. (**G**–**J**) The total placental area (**G**), labyrinth area (**H**), spongiotrophoblast area (**I**), and labyrinth/spongiotrophoblast ratio (**J**) were not significantly changed by placental hsFLT1 at 18.5 dpc. Data are presented in a box plot with median, interquartile range, and lower/upper extremes. n = number of placentas. * *p* ≤ 0.05 as determined by the Mann–Whitney test. Scale bar: 150 µm. MT = mesometrial triangle, D = decidua, Sp = spongiotrophoblast, L = labyrinth.

The area of the total placenta, the labyrinth, and spongiotrophoblast compartments were measured in placental sections stained with Masson–Goldner trichrome (MGT) to evaluate the impact of placenta-specific hsFLT1 overexpression on placental morphology (Figure 3A,F). At 14.5 dpc, the total placental area as well as the spongiotrophoblast area was enlarged in the male and female FGR groups compared to their controls (Figure 3B,D). The size of the labyrinthine area did not change (Figure 3C). The labyrinth/spongiotrophoblast quotient was reduced in both FGR groups with a significant reduction only in males (Figure 3E). At 18.5 dpc, no significant changes in total placental, labyrinth, or spongiotrophoblast area were detectable between FGR and Ctrl placentas (Figure 3F–J).

### 2.4. Elevated Placental hsFLT1 Levels Led to Increased Maternal Vascularization in the Labyrinth at 14.5 dpc

**Figure 4:**
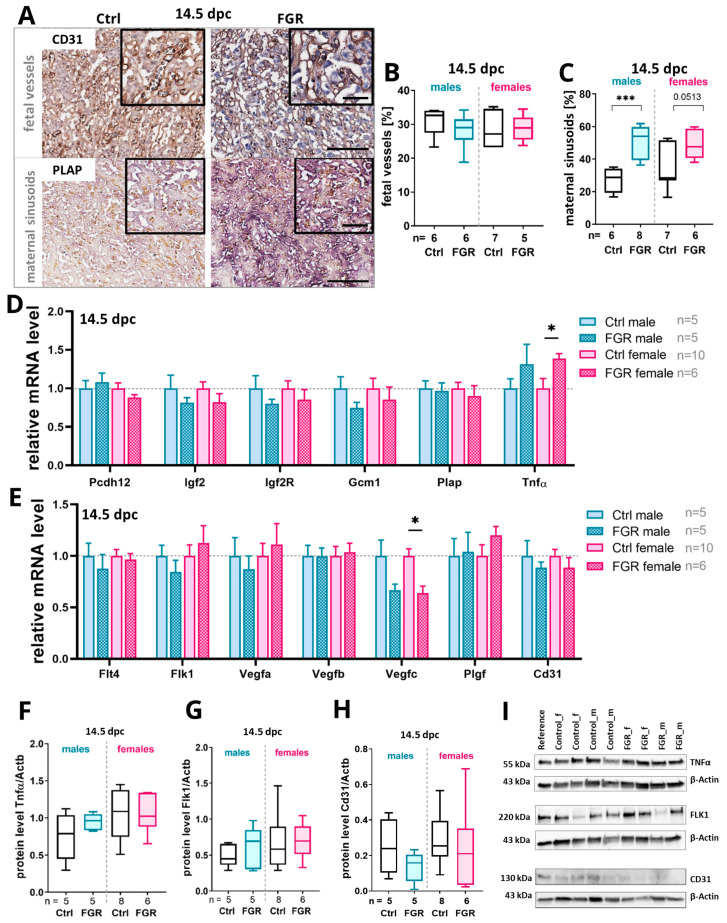
Impact of placenta-specific hsFLT1 overexpression on fetal and maternal vascularization at 14.5 dpc. (**A**) Immunohistochemical staining for Cd31 to indicate fetal vessels and placental alkaline phosphatase (PLAP) staining showed maternal sinusoids in Ctrl and FGR labyrinth compartments. (**B**) The fetal vessel area was not affected by hsFLT1 expression. (**C**) The percentage of maternal sinusoids was increased in the FGR groups compared to Ctrls, but only significantly in males. (**D**) The mRNA levels of *Igf2*, *Igf2R*, and *Gcm1* were downregulated in FGR placentas compared to controls. Expression of *Pcdh12* and *Plap* did not differ between groups while *Tnfα* expression was upregulated in both sexes of the FGR groups compared to controls. (**E**) The mRNA levels of *Flt4*, *Flk1*, *Vegfa*, *Vegfc*, and *Cd31* were downregulated in the male FGR group upon placenta-specific hsFLT1 expression. *Vegfc* expression was significantly decreased in the female FGR group compared to female controls. *Flk1*, *Vegfa*, and *Plgf* mRNA levels were upregulated in the female FGR group compared to the female Ctrl group. (**F**) The protein level of TNFα did not changed. (**G**) The protein level of FLK1 was slightly increased in both FGR groups compared to Ctrls. (**H**) The protein level of CD31 was reduced in the male and female FGR groups compared to respective Ctrls. The mRNA levels were determined by qPCR and normalized to the housekeeping gene *Gapdh*. (**I**) Representative blot images for TNFα, FLK1, CD31, and housekeeping gene β-actin. The mRNA levels were determined by qPCR and normalized to the housekeeping gene *Gapdh*. The protein levels were determined by Western blot analysis and normalized to the housekeeping gene β-actin. Data are presented in a box plot with median, interquartile range, and lower/upper extremes or in a bar graph with standard error. n = number of placentas. * *p* ≤ 0.05 and *** *p* ≤ 0.001, as determined by the Mann–Whitney test. Statistical analyses of correlation were calculated by Spearman r test. Scale bar = 150 µm; small scale bar = 50 µm.

As the morphological changes in the placental compartments were only visible at 14.5 dpc, a deeper analysis of the placenta was performed at this stage (Figure 4). At first, the effect of placenta-specific expression of hsFLT1 on placental vascularization in the labyrinth was investigated by measuring the expression of vessel markers such as CD31 for fetal vessels and placental alkaline phosphatase (PLAP) for maternal sinusoids (Figure 4A–C). The percentage of fetal vessels did not change at 14.5 dpc. However, the percentage of maternal sinusoids significantly increased in FGR placentas of both sexes compared to the Ctrls (Figure 4B,C). No significant correlation between maternal sinusoids or fetal vessels and the size of the labyrinth was detected (Figure S1P,Q).

There were no differences in key placental differentiation markers such as *protocadherin 12* (*Pcdh12*), *insulin-like growth factor 2* (*Igf2*), *Igf2 receptor* (*Igf2r*), *glia cell missing transcription factor 1* (*Gcm1*), and *Plap* between the groups and sexes at the mRNA level (Figure 4D). No significant changes could be detected between the groups and sexes of the fetuses except for a tendency for decreased expression of *Igf*, *Igf2r*, and *Gcm1* at 14.5 dpc. Remarkably, the mRNA expression level of the cytokine *tumor necrosis factor α* (*Tnfα*) was upregulated in FGR placentas of both fetal sexes but only significantly in female placentas compared to their respective controls (Figure 4D). The upregulation of TNFα protein levels was only present in the placentas of the male FGR group (Figure 4F,I).

Important angiogenesis markers of the vascular endothelial growth factor (VEGF) signaling pathway were screened to investigate the impact of placenta-specific hsFLT1 overexpression on placental vascularization. *Fms-like tyrosine kinase receptor 4* (*Flt4*) and *fetal liver kinase 1* (*Flk1*) as well as their ligands *placental growth factor* (*Plgf*), *Vegfa*, *Vegfb*, and *Vegfc* were analyzed. The expression level of most of the analyzed factors tended to be reduced in the male FGR group compared to the respective Ctrl group (Figure 4E) at day 14.5 dpc. The placentas of the female FGR group showed a significantly decreased *Vegfc* mRNA level compared to the Ctrl. In contrast, *Flk1*, *Vegfa*, and *Plgf* mRNA levels were upregulated in female FGR placentas. The analyses at the protein level showed slightly upregulated FLK1 levels in the FGR groups of both sexes (Figure 4H,I). FLT4 protein was not affected by placenta-specific hsFLT1 overexpression (Figure S2A). The endothelial cell marker *Cd31* was downregulated at the mRNA and protein levels in FGR placentas compared to their respective Ctrls for both sexes (Figure 4E,H,I).

### 2.5. Placenta-Derived hsFLT1 Led to a Reduced Amount of Fetal Labyrinthine Vessels and to an Accumulation of TNFα at 18.5 dpc

**Figure 5:**
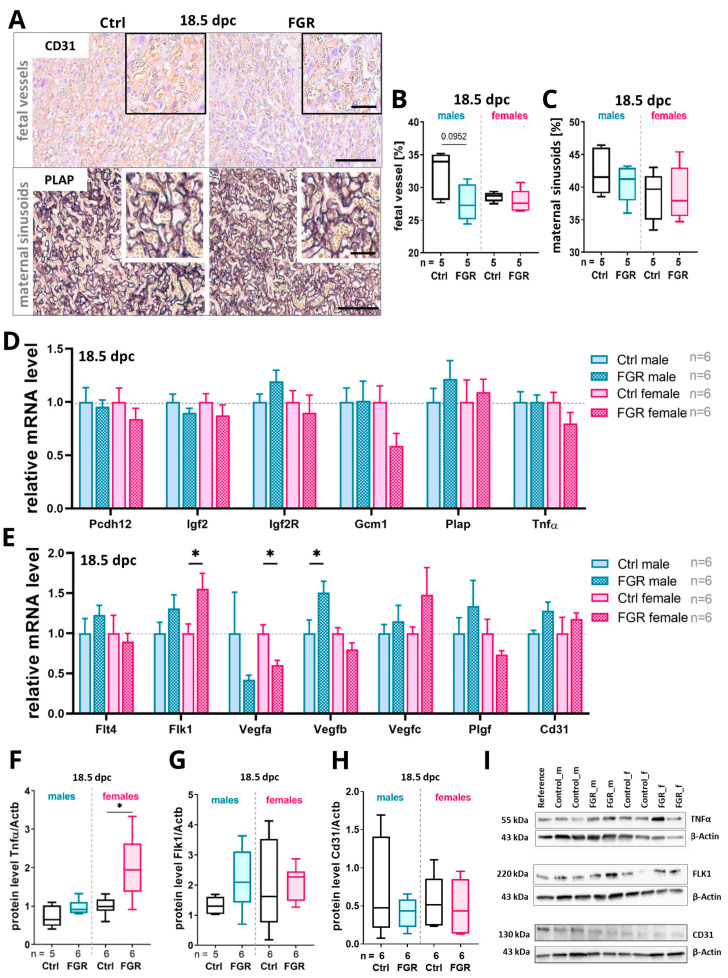
Impact of placenta-specific hsFLT1 overexpression on fetal and maternal vascularization at 18.5 dpc. (**A**) Immunohistochemical staining for Cd31 to indicate fetal vessels and PLAP staining shows maternal sinusoids. (**B**,**C**) The percentage of fetal vessels (**B**) was reduced by hsFLT1 expression and was more pronounced in male FGR placentas, whereas the percentage of maternal sinusoids (**C**) was not different in the FGR group compared to their respective Ctrls. (**D**) The mRNA expression of *Igf2R* and *Plap* was increased in male FGR placentas whereas *Gcm1* was decreased in female FGR placentas compared to the respective controls. The expression of *Pcdh12*, *Igf2*, and *Tnfα* did not differ between groups. (**E**) The mRNA levels of *Flk1*, *Vegfb*, and *Vegfc* were increased in the FGR group upon placenta-specific hsFLT1 expression compared to the Ctrls. The *Vegfa* expression was significantly decreased in the female FGR group compared to the female Ctrl. The expression of *Flt4*, *Plgf*, and *Cd31* did not change between the groups. (**F**) The TNFα protein level was significantly increased in female FGR placentas compared to controls. (**G**) The protein level of FLK1 was increased in both FGR groups compared to Ctrls. (**H**) The protein level of CD31 was not affected by placental hsFLT1 expression. (**I**) Representative blot images for TNFα, FLK1, CD31, and housekeeping gene β-actin. The mRNA levels were determined by qPCR and normalized to the housekeeping gene *Gapdh*. The protein levels were determined by Western blot analysis and normalized to the housekeeping gene β-actin. Data are presented in a box plot with median, interquartile range, and lower/upper extremes or in a bar graph with standard error. n = number of placentas. * *p* ≤ 0.05as determined by the Mann–Whitney test. Statistical analyses of correlation were calculated by Spearman r test. Scale bar = 150 µm; small scale bar = 50 µm.

Because vascularization of the placental labyrinth was affected by placenta-specific hsFLT1 overexpression, this compartment was further analyzed at 18.5 dpc in detail (Figure 5). The percentage of fetal vessels was reduced in both sexes but the reduction was more pronounced in males. Regarding the maternal sinusoids, only the female FGR group showed a decrease in percentage compared to the respective Ctrl (Figure 5A–C). The percentage of fetal vessels trended towards a correlation (*p* = 0.0876) with the labyrinthine area in male FGR placentas but not in female placentas (Figure S1R), whereas the percentage of maternal sinusoids did not correlate with the size of the labyrinthine area in both sexes (Figure S1S).

Regarding the transcript expression of markers for trophoblast differentiation at 18.5 dpc, *Igf2r* was upregulated in the male FGR group and downregulated in the female FGR group compared to their respective controls. Further, *Gcm1* was downregulated only in females of the FGR group at 18.5 dpc. The mRNA expression of *Plap* was upregulated in the FGR group in both sexes at 18.5 dpc (Figure 5D). *Tnfα* mRNA expression showed a trend towards a decrease in FGR placentas of female fetuses (Figure 5D), but TNFα protein levels were increased in both sexes of the FGR group, with a significant increase seen in females (Figure 5F,I). *Flk1* mRNA expression was significantly upregulated and *Vegfa* was significantly decreased in female FGR placentas. In male FGR placentas, *Vegfb* was significantly increased (Figure 5E). *Vegfc* trended towards an increase in the FGR group, and was most pronounced in females. FLK1 protein was found to be trending towards an increase in the FGR group (Figure 5G,I), whereas the CD31 protein level did not change (Figure 5H,I).

It is known from several studies from our group and others in mice and humans that sFLT1 is regulated by hypoxia [15,16,17,18]. Therefore, we analyzed the impact of placental hsFLT1 overexpression on the mRNA expression levels of hypoxia pathway markers such as *heme oxygenase 1* (*Ho1*), *hypoxia-inducible factors* (*Hifs*), and *prolyl hydroxylases* (*Phds*) (Figure S2C,D). At 14.5 dpc, a trend towards an increase in *Hif2α* (*p* = 0.09) and a significant upregulation of *Phd2* was detected in the female FGR group compared to the controls. A significant decrease in *Phd2* in the male FGR groups was observed (Figure S2C). At 18.5 dpc, *Hif2α* expression was significantly increased in male FGR placentas, but not in female placenta, and *Phd1* was upregulated in the female FGR group (Figure S2D).

### 2.6. Placental hsFLT1 Levels Reduced the Expression of Placental Nutrient Transporters and Glycogen Cells in the Spongiotrophoblast

**Figure 6:**
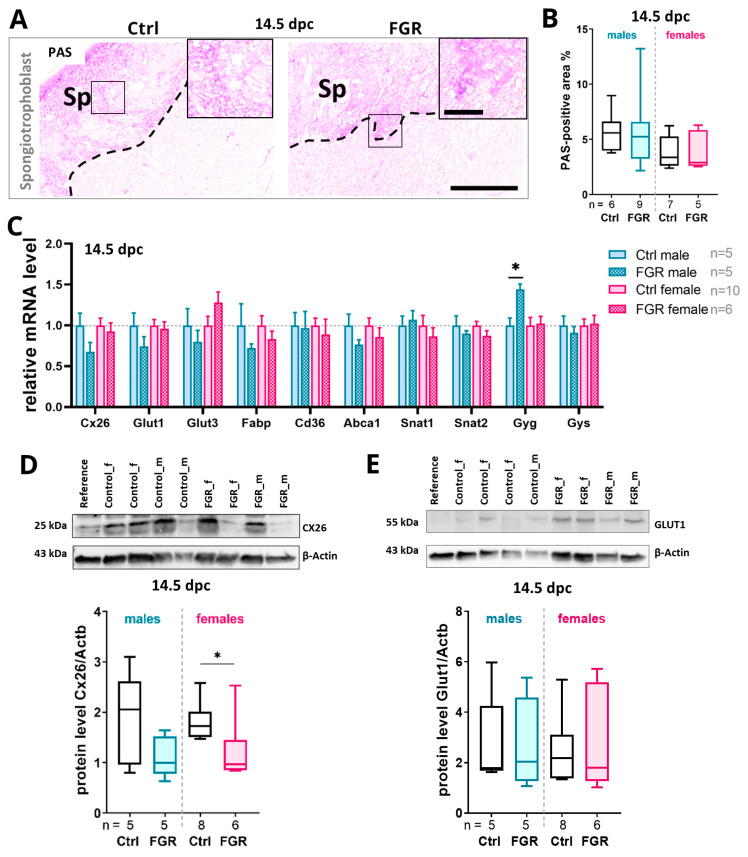
Impact of placenta-specific hsFLT1 expression on glycogen storage and nutrient transporter expression in the placenta at 14.5 dpc. (**A**) Representative images of periodic acid Schiff (PAS)-stained placentas with a focus on the spongiotrophoblast layer in the Ctrl and FGR groups. Arrows indicate glycogen cell-enriched areas. (**B**) Quantification of the PAS-positive area in the spongiotrophoblast layer showed no difference between area of glycogen-containing cells in both groups. (**C**) The mRNA expression levels of most of the analyzed nutrient transporters in the placenta were downregulated upon hsFLT1 expression. The *Glut3* mRNA level was trending towards an increase by trend in the female FGR group and *Gyg* mRNA was significantly upregulated in the male FGR group compared to its respective Ctrl. (**D**) The CX26 protein level was reduced in both FGR groups with a significant difference in the female FGR group. (**E**) The protein level of GLUT1 was upregulated in both sexes of the FGR group compared to Ctrls. The mRNA levels were determined by qPCR and normalized to the housekeeping gene *Gapdh*. The protein levels were determined by Western blot analysis and normalized to the housekeeping gene β-actin. Data are presented in a box plot with median, interquartile range, and lower/upper extremes or in a bar graph with standard error. n = number of placentas. * *p* ≤ 0.05 as determined by the Mann–Whitney test. Scale bar = 400 µm; small scale bar = 150 µm.

To evaluate the impact of placenta-specific hsFLT1 expression on nutrient exchange and storage in the placenta, the percentage of the area with glycogen-containing cells within the spongiotrophoblast layer was evaluated by periodic acid Schiff (PAS) staining and the expression levels of important nutrient transporters for glucose, fatty acid, and amino acids were measured. At 14.5 dpc, the area of glycogen-containing cells in the spongiotrophoblast in the FGR groups was comparable to that of the Ctrl groups (Figure 6B). Of note, *glycogenin 1* (*Gyg*) mRNA levels were upregulated in the male FGR group (Figure 6C). The mRNA level of the gap junction protein *connexin 26* (*Cx26*) and *glucose transporters 1* and *3* (*Glut1*/*Glut3*) were downregulated in the male FGR group compared to its respective Ctrl group (Figure 6C). Along with the mRNA level, the protein level of Cx26 was decreased in both sexes of the FGR group, but only significantly in females (Figure 6D). No difference in the protein levels of GLUT1 could be detected between Ctrl and FGR groups at 14.5 dpc (Figure 6E).

**Figure 7:**
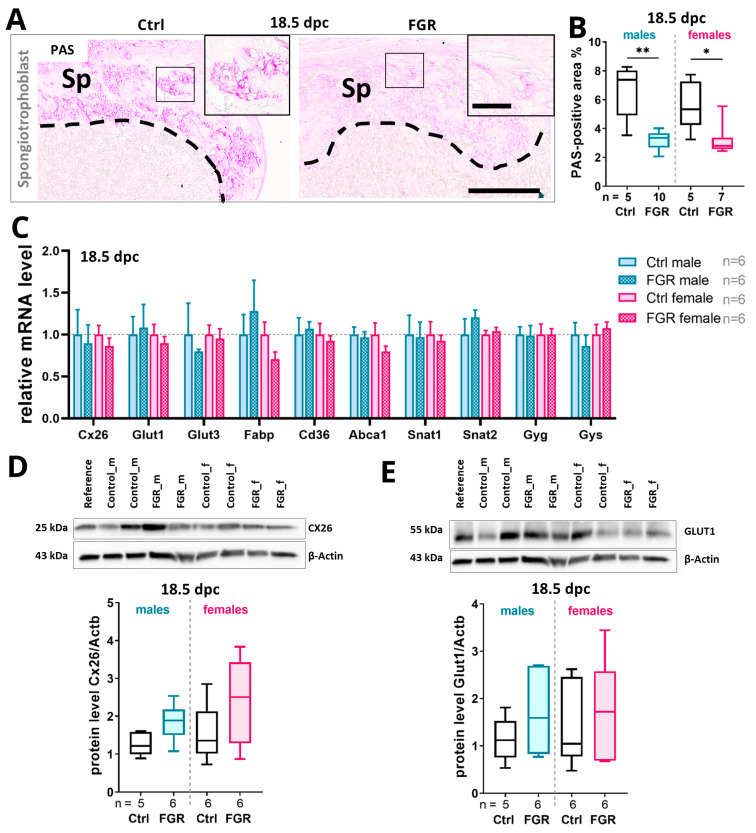
Impact of placenta-specific hsFLT1 expression on glycogen storage and nutrient transporter expression in the placenta at 18.5 dpc. (**A**) Overview of the spongiotrophoblast layer in PAS-stained placentas of Ctrl and FGR groups. Arrows indicate glycogen cell-enriched areas. (**B**) PAS-positive area in the spongiotrophoblast layer was reduced in both FGR groups compared to controls, with males being more affected than females. (**C**) The expression levels of most of the analyzed nutrient transporters in the placenta were not changed upon hsFLT1 expression. (**D**,**E**) Both glucose transporter proteins (Cx26 and GLUT1) were trending towards an increase in protein levels in male and female placentas of the FGR group compared to Ctrls. The mRNA levels were determined by qPCR and normalized to the housekeeping gene *Gapdh*. The protein levels were determined by Western blot analysis and normalized to the housekeeping gene β-actin. Data are presented in a box plot with median, interquartile range, and lower/upper extremes or in a bar graph with standard error. n = number of placentas. * *p* ≤ 0.05 and ** *p* ≤ 0.01 as determined by the Mann–Whitney test. Scale bar = 400 µm; small scale bar = 150 µm.

At 18.5 dpc, a significant decrease in the area with glycogen-containing cells in the spongiotrophoblast was observed in both FGR groups (Figure 7A,B). The correlation analyses showed that the PAS-positive glycogen cell area in the placenta was significantly correlated with the fetal weight of the male FGR fetuses, but not that of the female fetuses (Figure S2B). No significant changes in the transcript expression levels of the nutrient transporters were found among the groups at 18.5 dpc (Figure 7C). However, we observed a trend towards increases in Cx26 and GLUT1 protein levels in the FGR placentas compared to the respective Ctrls (Figure 7D,E).

## 3. Discussion

We generated and analyzed a murine model overexpressing the clinical biomarker for preeclampsia, human sFLT1, in a spongiotrophoblast-specific manner. Low levels of the anti-angiogenic protein sFLT1 were detected in the serum of the dams, leading to characteristic malformations of the placenta, such as an increased spongiotrophoblast layer, impaired fetal and maternal vascularization in the labyrinth, reduced placental glycogen storage, reduced nutrient transporter expression, as well as signs of hypoxic and inflammatory dysregulation. Overall, these alterations affected fetal growth, leading to FGR. As such, our study clearly demonstrates that comparably low levels of sFLT1 are sufficient to interfere with the well-orchestrated development of the placenta, leading to disturbances to not only placental development, but also fetal growth. 

The effect of placenta-specific hsFLT1 overexpression on feto-placental development in the transgenic hsFLT1/tTA/Tpbpa-Cre mouse model mimics the pathophysiological processes of the human pregnancy disorder PE. In this model, hsFLT1 expression was exclusively upregulated in spongiotrophoblast cells starting from midgestation at 8.5 dpc due to the *Tpbpa*-promoted Cre expression. Thus, the expression pattern is spatially and temporally similar to human PE, since it is assumed that sFLT1 is overexpressed by the placenta starting around the 20th week of gestation during human PE [1,19].

Our study showed that upon placental overexpression of hsFLT1, the fetal weight of both sexes was significantly reduced at the end of pregnancy at 18.5 dpc. The undetected FGR phenotype at 14.5 dpc might be explained by the short interval between the intrinsic start of the hsFLT1 expression (8.5 dpc) and the time point 14.5 dpc; thus, the exposure of the fetus to low hsFLT1 levels at 14.5 dpc might not be sufficient to impair overall fetal development. Nonetheless, a significant impact of placenta-derived hsFLT1 reduced the fetal body and organ weights at term. Furthermore, the mRNA expression of *hsFLT1* was increased in male FGR placentas compared to female placentas, further emphasizing the sex-specific differences in this model, showing a stronger impairment in males. This was previously shown in several studies where pregnancies with male fetuses were more prone to early miscarriage, pregnancy-induced hypertension, and spontaneous preterm birth [20,21,22]. In a recent study by Kedziora et al., 2022 [23], it was revealed that the fetal sex has a prominent effect on the placental transcriptome in diabetic pregnancies which strengthen the finding that the placenta is a sexually dimorphic organ. Surmon et al. (2012) [24] showed that in a normal murine pregnancy, female placentas express higher levels of murine sFLT1 than male placentas. Therefore, males may have a lower ability to adapt to the increased sFLT1 levels and suffer more from the placenta-specific sFLT1 overexpression.

The functionality of the hsFLT1/tTA/Tpbpa-Cre mouse model was confirmed by the detection of elevated hsFLT1 serum levels in dams and significantly increased expression levels exclusively in the placenta at 14.5 dpc and at term (18.5 dpc). Because the hsFLT1 expression is limited to the spongiotrophoblast, hsFLT1 serum concentrations were about three times lower at term compared to previously analyzed hsFLT1 levels in the lentiviral-based placenta-specific sFLT1 mouse models, where hsFLT1 is expressed by the whole placenta [5,8]. The highest sFLT1 serum levels were achieved in mice that systemically overexpress sFLT1 through adenoviral transduction [25] or the transgenic hsFLT1 PE/FGR mouse model established by our group [14,15]. Even if the transgenic hsFLT1/tTA/Tpbpa-Cre mice described here did not reach the high hsFLT1 serum levels that were described previously, our model more specifically simulates the human disease of PE and even the low levels of spongiotrophoblast-derived hsFLT1 are sufficient to show a significant impact on fetal and placental development.

### 3.1. The Placental Spongiotrophoblast Compartment Is Strongly Affected by Placenta-Specific hsFLT1 Expression at 14.5 dpc

The spongiotrophoblast layer consists of trophoblast giant cells, spongiotrophoblast cells, and glycogen cells and is mainly involved in hormone secretion and metabolism [26]. Our study revealed that the spongiotrophoblast area was significantly increased in size at 14.5 dpc, which was not observed in other sFLT1 mouse models [8,14,25]. As hsFLT1 is expressed by cells of the spongiotrophoblast layer, the enlargement of this area might be a consequence of the increased expression in this particular tissue. Spongiotrophoblast defects are often observed in transgenic mouse models of gestational diseases and the outcomes of the fetuses vary depending on the manipulated genes and on the form of manipulation in the model [27]. Enlarged spongiotrophoblast layers and increased numbers of spongiotrophoblast or glycogen cells due to various genetic mutations are often associated with FGR fetuses [28,29,30]. The expansion could also be a compensating mechanism to fulfil the original functions of secreting hormones to maintain pregnancy and providing energy reserves by accumulating glycogen to counteract the fetal growth restriction and thereby improve fetal growth. An indicator for an uncontrolled proliferation of spongiotrophoblast cells in our model could be the inhomogeneous border between the labyrinth and spongiotrophoblast layers in the FGR placentas, which was observed during microscopic evaluation. This observation could hint towards mislocalized glycogen and spongiotrophoblast cells and represents a structural dysmorphology that is often found in other transgenic mouse models with FGR [31,32].

### 3.2. Placenta-Specific hsFLT1 Expression Leads to Reduced Expression of Glucose Transporters and Reduced Placental Glycogen Storage

Even though the size of the labyrinthine area was not significantly reduced upon placenta-specific hsFLT1 expression, a malfunction of the placental labyrinth cannot be excluded. The labyrinth compartment is indispensable for providing the fetus with nutrients by transporting them from the maternal blood into the fetal circulation [33]. The protein level of the gap junction protein CX26 was found to be significantly reduced in FGR placentas at 14.5 dpc. The importance of CX26 for glucose uptake into the fetal compartment and thus for fetal growth was shown in studies on *Cx26* knock-out mice by Gabriel et al., 1998 [34]. The consequences of lacking CX26 in the placenta were reduced fetal weight and prenatal lethality due to a deficiency in glucose, which is the main nutrient to ensure fetal growth. Therefore, the upregulated CX26 protein level later at 18.5 dpc in mice with placenta-specific hsFLT1 overexpression was presumably a counterreaction since CX26 is indispensable for fetal survival. At term, we also detected a reduced number of glycogen cells in the spongiotrophoblast layer of FGR placentas, with the strongest effect seen in males. Similar findings in regard to the decreased numbers of glycogen cells were found in Kühnel et al. 2017 [8] in the lentiviral-induced sFLT1 placentas (but without the sex differences). The reduced presence of glycogen cells at term in FGR mice of both sexes hints towards disrupted glucose transport and storage mechanisms in these placentas. Besides a reduced level of Cx26 mRNA, the lentiviral mouse model showed low mRNA levels of *insulin-like growth factor 2* (*Igf2*) [8]. The main source of IGF2 in rodents is the placenta. IGF2 controls the availability of nutrients by regulating the diffusional exchange during placental development and an absence of IGFs in the murine placenta leads to a FGR phenotype [35,36]. This could not be verified for our transgenic mouse model. However, the molecular mechanisms through which IGF2 regulates the nutrient supply are not completely understood yet. The *Igf2* expression level at 14.5 dpc showed only a tendency towards a downregulation which could be linked to the reduced *Cx26* expression as the expression of *Igf2*, similar to *Cx26*, starts at around 9.5 dpc in spongiotrophoblast cells [35,37].

### 3.3. Placenta-Derived hsFLT1 Leads to Increased Hypoxia, Inflammation, and Disturbed Vascularization at the Feto-Maternal Interface Especially in Males

Our study revealed that at 14.5 dpc, the percentage of maternal sinusoids was increased in FGR placentas while the amount of fetal vessels was decreased at the feto-maternal interface in the labyrinthine layer. In the PE/FGR hsFLT1 mouse model, disturbed labyrinthine vascularization was observed as well [14,15]. Furthermore, the maternal sinusoid/fetal vessel ratio was significantly increased in FGR placentas due to the significantly reduced fetal vessels in the labyrinth. Here, males were also affected to a greater degree than females. Both findings from different sFLT1 mouse models, in Vogtmann et al., 2021 [15], and in this study, suggest that an increased ratio of maternal sinusoids to fetal vessels in the labyrinth is a sign of dysregulated vascularization triggered by hsFLT1. Further, this dysregulation gives rise to abnormal placental and fetal development and FGR.

The increased percentage of maternal sinusoids was measured by staining for placental alkaline phosphatase (PLAP), an enzyme expressed by syncytiotrophoblast cells. These cells form the main part of the placental barrier and an increase in placental barrier thickness can inhibit the passive diffusion from the maternal to fetal vascular system, which could also contribute to a decreased passive diffusion rate of oxygen over the placenta [36,38]. Increased hypoxic conditions are characteristics for placental maladaptation in preeclampsia and fetal growth restriction [39,40]. Additionally, we detected increased expression of the hypoxic factor *Hif2α* in placentas with placental hsFLT1 overexpression. These results are in line with our previous findings in mice systemically overexpressing hsFLT1, where hypoxic conditions in the placenta were confirmed by use of the hypoxic marker pimonidazole and protein analysis of heme oxygenase 1 (HO1) [16]. The expression of *HIF2α* was found to be linked to the upregulation of *FLT1* gene expression in human placental trophoblast cells in vitro [41]. Conversely, we were able to show that hsFLT1 is also capable of upregulating *Hif2α* gene expression in vivo [15].

The mice with placenta-specific hsFLT1 overexpression also showed signs of increased inflammation, as the expression of TNFα was upregulated by placental hsFLT1. The pro-inflammatory cytokine TNFα is expressed and secreted physiologically from syncytiotrophoblast cells in the placenta [42], although upregulated placental TNFα expression in pregnant women is associated with FGR [43]. Here, in this study, the *Tnfα* mRNA level was upregulated in FGR placentas at 14.5 dpc, resulting in elevated protein levels at 18.5 dpc due to placental hsFLT1 overexpression. It was recently discovered that, at term, human placental villi TNFα signaling is mediated by sphingosine kinase 1 (SPHK1) and this signaling pathway is reinforced by increased TNFα levels in preeclamptic placentas [44]. Similar to the results presented here, in our prior investigation in the PE mouse model with systemic hsFLT1 overexpression, an increase in *Tnfα* transcript expression was observed and was accompanied by an FGR phenotype as well [14,15], strengthening the connection between the expression of TNFα in the placenta and elevated serum hsFLT1 levels with placental inflammation. Increased levels of TNFα in the placenta and serum in pregnancy are associated with preeclampsia, as had been shown by Aggarwal et al. (2019) [45] and Wang and Walsh (1996) [46].

## 4. Materials and Methods

### 4.1. Generation of hsFLT1/tTA/Tpbpa-Cre Mice and Experimental Setting

Three mouse lines were used to generate the hsFLT1/tTA/Tpbpa-Cre mouse strain: hsFLT1 mice (Col1A1^tm2(tetO-FLT1*)Hsc^ (MGI: 6202353); refer to [14] for detailed information regarding the generation of the hsFLT1 mouse strain), tTA mice (Gt(ROSA)26Sort^m1(tTA)Roos/^J (JAX stock #008600, The Jackson Laboratory, Bar Harbor, ME, USA)), and Tpbpa-Cre mice [26].

Mating was performed overnight and the following day was counted as 0.5 dpc. The mating strategy is depicted in Figure 1A. The parental generation of the double-transgenic hsFLT1/tTA and hsFLT1/Tpbpa-Cre mice are not able to express hsFLT1. By combining all three transgenic alleles in the first filial generation, the induction of placenta-specific hsFLT1 expression was enabled (Supplement Figure S1A). The placenta-specific expression of the Cre recombinase is regulated by the spongiotrophoblast-specific protein alpha (Tpbpa) promoter which is active from 8.5 days post conception (dpc) onwards. Therefore, the expression of hsFLT1 is temporally and spatially regulated by the intrinsic expression of Cre. Due to the experimental design, hsFLT1 expression only occurred in triple-transgenic placentas (FGR group). In the control group (Ctrl), double-transgenic hsFLT1/tTA mice lacking the Tpbpa-Cre allele were mated and therefore were incapable of hsFLT1 expression. Dams were sacrificed and samples were collected at 14.5 dpc (Ctrl: n = 3, FGR: n = 4) and 18.5 dpc (Ctrl: n = 3, FGR: n = 5). The fetuses and corresponding placentas were subdivided directly after preparation and randomly assigned for histological and molecular biological analyses in a 50:50 ratio. For each experiment, 1–4 fetuses/placentas of each sex were used per dam. The specific number of dams/fetuses/placentas used for each experiment is given in each figure. Mice were housed in a specific pathogen–free environment at the animal facility of the University Hospital Essen, were exposed to cycles of 12 h of light/dark, and were provided with food and water ad libitum.

### 4.2. Tissue Preparation

At 14.5 dpc or 18.5 dpc, pregnant mice were killed by cervical dislocation after narcotizing with 5% isoflurane. The heart was punctured and maternal blood was collected. Fetuses, placentas, and maternal organs were removed and washed in sterile phosphate-buffered saline (PBS). The samples were weighed using an ALJ 220-4 NM analytical balance (Kern, Ebingen, Germany) with a linearity of ±0.2 mg. Half of the fetuses and placentas were frozen at −80 °C for molecular biological analyses and the maternal mesometrial triangle (MT) tissues with most parts of the decidua were separated from the fetal placenta. For histological analyses, the other half of the samples were fixed in 4% paraformaldehyde (PFA) for 24 h and transferred to 70% ethanol to store until embedding in paraffin.

### 4.3. Genomic DNA Isolation, Genotyping, and Sex Determination

The genomic DNA of fetuses was isolated from tail tips with the REDExtract-N-AmpTM Tissue PCR Kit (Sigma-Aldrich, St. Louis, MO, USA) according to the manufacturer’s instructions. A BioPhotometer Plus (Eppendorf, Hamburg, Germany) was used to quantify the DNA. A standard PCR program was used to determine the sex and genotype of mice: hsFLT1: initial denaturation, 95 °C for 5 min; 40 cycles of 94 °C for 45 s, 60 °C for 45 s, 72 °C for 1 min; final extension, 72 °C for 5 min. tTA: initial denaturation, 94 °C for 5 min; 30 cycles of 94 °C for 1 min, 60 °C for 1 min, 72 °C for 2 min; final extension, 72 °C for 10 min. Cre: initial denaturation, 94 °C for 3 min; 35 cycles of 94 °C for 30 s, 61 °C for 1 min, 72 °C for 1 min; final extension, 72 °C for 2 min. Sex: initial denaturation, 95 °C for 4.5 min; 35 cycles of 95 °C for 35 s, 50 °C for 1 min, 72 °C for 1 min; final extension, 72 °C for 5 min. The respective primers used for genotyping were used in the PCRs (see Supplementary Table S1).

### 4.4. RNA Extraction, cDNA Synthesis, and Quantitative PCR

RNA was isolated from 20 mg of frozen tissue with an AllPrep DNA/RNA/Protein Mini Kit (Qiagen, Venlo, The Netherlands) according to the manufacturer’s instructions. Approximately 2 µg of RNA was transcribed into complementary DNA (cDNA) by reverse transcription (RT) as previously described [8]. Quantitative real-time PCR (qPCR) was performed to measure gene expression from placental tissue. The primer sequences are listed in Supplementary Table S1. A total of 1 µL of cDNA with 19 µL of PowerUP SYBR Green Master Mix (#A25742; Applied Biosystems, Foster City, CA, USA) were analyzed in triplicates using the ABI Prism 7300 Sequence Detection System (Applied Biosystems, Foster City, CA, USA) with a standard PCR program. The standard curve method was used to quantify the gene expression. Each sample was normalized to the housekeeping gene glyceraldehyde-3-phosphate dehydrogenase (Gapdh) and gene expression in the male and female FGR groups were normalized to their respective Ctrl group.

### 4.5. Analysis of hsFLT1 Serum Levels

Maternal blood was coagulated at room temperature for 2 h. The clotted blood was centrifuged at 3000× *g* and 4 °C for 15 min, and the serum was collected and stored at −80 °C. For measurements of serum hsFLT1 concentrations, a BRAHMS KRYPTOR compact PLUS analyzer (Thermo Fischer Scientific, Waltham, MA, USA) was used. The BRAHMS sFlt-1 KRYPTOR assay was performed according to the manufacturer’s instructions. The detectable concentration limit is between 22 pg/mL and 90,000 pg/mL.

### 4.6. Histological and Morphometrical Analysis

Placentas were fixed with 4% PFA, dehydrated in ethanol, and embedded in paraffin. Sections (5 µm thick) were cut using a microtome and mounted on either Superfrost Plus Slides (R. Langenbrinck, Emmendingen, Germany) for immunohistochemistry or standard slides (Engelbrecht Medizin- und Labortechnik GmbH, Edermünde, Germany) for Masson–Goldner trichrome (MGT), periodic acid Schiff (PAS), and placental alkaline phosphatase (PLAP) staining.

Digitalization of the stained slides was conducted by the West German Biobank, University Hospital Essen. The slides were scanned with an Aperio ScanScope AT2 (Leica, Wetzlar, Germany) at 40× magnification in transmission mode. Digital images were converted into TIFF files using Image Scope (Version 12.3.3.5048, Leica) and analyzed with ImageJ/FIJI (Version 1.53t) [47]. Morphometric analyses were performed on placenta sections in the region of the umbilical cord.

### 4.7. Staining with Masson–Goldner Trichrome (MGT)

For morphometric analyses of the placental compartments, slides were deparaffinized, rehydrated, and stained with an MGT staining kit (Carl Roth, Karlsruhe, Germany) according to the manufacturer’s instructions. After staining, the slides were dehydrated and mounted with xylene mountant. Three slides from different sections of each placenta (approximately 100 µm apart) were analyzed to measure the area of the labyrinth and spongiotrophoblast layer with ImageJ (Version 1.53t).

### 4.8. Staining with Periodic Acid Schiff Reagent (PAS)

The PAS reaction is used to visualize glycogen-storing cells in the spongiotrophoblast layer and was described previously in detail [15]. In short, deparaffinized and rehydrated slides were incubated with 1% periodic acid (Carl Roth, Karlsruhe, Germany) for 10 min, incubated with Schiff’s reagent (Carl Roth, Karlsruhe, Germany) for 20 min, and then probed with sulfite water for 6 min (10% sodium bisulfite solution, 1 M HCl) to reduce the pseudo-PAS reaction. The slides were dehydrated, mounted with xylene mountant, and digitalized as described above. TIFF files were converted to binary images to quantify the PAS-positive area of the spongiotrophoblast (three sections per placenta) with ImageJ (Version 1.53t).

### 4.9. Staining for Placental Alkaline Phosphatase (PLAP)

This staining was used to detect the maternal sinusoids in the placental labyrinth. The slides were de-waxed and rehydrated. The sections were probed with Nitro Blue Tetrazolium (NBT)/5-bromo-4-chloro-3-indolyl phosphate (BCIP) (Merck, Darmstadt, Germany) for 1 h. The stained slides were dehydrated, mounted, and digitalized as described above. Binary images were used to determine the PLAP-positive area of the labyrinth (three sections per placenta) with ImageJ (Version 1.53t).

### 4.10. Immunohistochemical Analysis

Immunohistochemistry was used to determine the percentage of CD31+ endothelial cells in the labyrinthine part of the placenta as a marker for fetal vessels. The detailed protocol was described previously [14]. Deparaffinized and rehydrated slides were boiled in citrate buffer to retrieve the antigens. The cell membrane was permeabilized with TritonX and H_2_O_2_/methanol was used to block endogenous peroxidase. The tissue sections were blocked with bovine serum albumin and incubated overnight at 4 °C with anti-CD31 antibody (Supplementary Table S2). Incubation with the biotinylated secondary antibody (Supplementary Table S2) and amplification of the signal was performed with an Vectastain Elite^®^ ABC Kit (Vector Laboratories, Burlingame, USA) according to the manufacturer’s instructions. Antigens were visualized with the Liquid DAB+ Substrate Chromogen System (Dako, Carpinteria, CA, USA). Dehydrated and mounted slides were digitalized as described and binary images were used to quantify the Cd31-positive area of the labyrinth (two sections per placenta) with ImageJ (Version 1.53t).

### 4.11. Immunoblot Analysis

Proteins from the placenta were isolated with the AllPrep DNA/RNA/Protein Mini Kit (Qiagen, Venlo, The Netherlands) according to the manufacturer’s instructions using 20 mg of frozen tissue. The protein concentration was measured with the Pierce BCA Protein Assay Kit (Thermo Scientific, Rockford, IL, USA) according to the manufacturer’s instructions. The protocol for the immunoblot analysis was described previously in detail [15]. In short, 20 µg of total protein lysate was separated on 4–15% polyacrylamide gels (Biorad, Hercules, CA, USA). The separated proteins were transferred onto a nitrocellulose membrane by a semi-dry transfer method. The membrane was incubated in 5% [*w*/*v*] milk powder solved in TBS-T to block non-specific binding sites. Primary antibodies specific for Cd31, CX26, VEGFR2/FLK1, VEGFR3/FLT4, GLUT1, TNFα, and β-actin were diluted in 0.5% [*w*/*v*] bovine serum albumin or 0.5% [*w*/*v*] milk powder in TBS-T peroxidase (Supplementary Table S2). After incubation with the primary antibody overnight at 4 °C, the membranes were washed and incubated at room temperature for 1 h with the corresponding secondary antibody (Supplementary Table S2). The SuperSignal West Dura Extended Duration Substrate Kit (Thermo Fisher Scientific, Pittsburgh, PA, USA) was used according to the manufacturer´s instructions to detect protein bands. Images were obtained with the Chemidoc XRS+ imaging system (BioRad, Feldkirchen, Germany). Specific bands, located through protein size with the PageRuler™ Prestained Protein Ladder (Thermo Fisher Scientific, Pittsburgh, PA, USA), were densitometrically analyzed with the Image Lab software (Version 6.0.0, BioRad Laboratories, Hercules, CA, USA) to determine the protein level. Normalization was performed against the housekeeping gene β-actin and immunoblots were standardized against the same reference sample that was run on each blot.

### 4.12. Statistical Analysis

Statistical differences between the Ctrl and FGR groups were calculated with the Mann–Whitney test (unpaired and non-parametric). The probability value (*p*-value) was marked with asterisk (*) for ≤0.05, ** for ≤0.01, and *** for ≤0.001. Associations between selected variables were tested with the Spearman correlation r test (two-tailed and non-parametric). Data are either presented as the mean value with standard error or in box plots with the median, interquartile range, and lower/upper extremes. Potential outliers were detected with the Grubbs Test (https://www.graphpad.com/quickcalcs/grubbs1/; accessed on 10 November 2023) and excluded from the calculations. All data were analyzed with GraphPad Prism software (Version 8.4.2, GraphPad, La Jolla, CA, USA).

## 5. Conclusions

This study investigated the effect of placenta-derived human sFLT1 overexpression starting at midgestation on fetal growth and placental function in a transgenic mouse model. Although the overexpression of the angiogenesis inhibitor sFLT1 was restricted to the spongiotrophoblast layer, it is sufficient to impair placental function and fetal growth. Additionally, sex-specific differences regarding the response to placental sFLT1 were detected, with males being more strongly affected by placental hsFLT1 than females. Using this mouse model, we will soon be able to test therapeutic options that target placental sFLT1 to improve fetal growth and unravel the role of placenta-derived hsFLT1 in the context of fetal growth restriction and preeclampsia.

## Data Availability

The authors declare that all supporting methods are available within the article. The data that support the findings of this study are available from the corresponding author on reasonable request.

## Supplementary Materials

The following supporting information can be downloaded at: https://www.mdpi.com/article/10.3390/ijms25042040/s1.
