# Supplementary material for: Overexpression of Human sFLT1 in the Spongiotrophoblast Is Sufficient to Induce Placental Dysfunction and Fetal Growth Restriction in Transgenic Mice
Source: Int J Mol Sci. 2024 Feb 7;25(4):2040. doi: 10.3390/ijms25042040 (PMC10888837; doi:10.3390/ijms25042040)
Supplement: Supplementary file 1 [file ijms-25-02040-s001.zip › ijms-2809635-supplementary.pdf]

Supplementary Table S1: Primers used for gene expression analysis, genotyping, and sex determination.

Gene	NCBI number	Primer sequence (5'→3')	Product length (bp)
**Genotyping**			
hsFLT1	NM_001159920.2	for: CAAGGACGTAACTGAAGAGG<br>rev: TTTCTTCCCACAGTCCCAAC	465
Col1a1	/	for: CCATCCCAACAATACATCACA<br>rev: TGGTTTCTTTGGGCTAGAGG	200
tTA	/	for: AAGGGAGCTGCAGTGGAGTA<br>rev-wt: CCGAAAATCTGTGGGAAGTC	297
**Sex determination**			
IL-3	NM_010556.4	for: GGGACTCCAAGCTTCAATCA<br>rev: TGGAGGAGGAAGAAAAGCAA	544
Sry	NM_011564.1	for: TGGGACTGGTGACAATTGTC<br>rev: GAGTACAGGTGTGCAGCTCT	402
**Reference/Housekeeping gene**			
Gapdh	XM_011241214.1	for: ACAACTCACTCAAGATTGTCAGCA<br>rev: ATGGCATGGACTGTGGTCAT	121
**Glucose and glycogen metabolism**			
Glut-1	NM_011400.3	for: GCTGTGCTTATGGGCTTCTC<br>rev: ACACCTGGGCAATAAGGATG	202
Glut-3	NM_011401.4	for: GGAGGAGAACCCTGCATATGATA<br>rev: TGGCTTCATAGTCATCCTTTAGTAAC	96
Gyg	NM_001355261.1	for: TATACTCCTACCTCCCGGCA<br>rev: TTGACAAGGCCATGGTGTTG	223
Gys1	NM_030678.3	for: CTGGCCGCTATGAGTTTTCC<br>rev: TGGGGTGTCCCATAGTTGT	194
Cx26	NM_008125.3	for: ATGCTACGACCACCACTTCC<br>rev: TACGGACCTTCTGGGTTTTG	194
**Fatty acid transporters**			
Cd36	NM_001159558.1	for: CAGTGCAGAAACAATGGTTGTCT<br>rev: TGACATTTGCAGGTCTATCTACG	137
Fabp	NM_010174.2	for: TTTGTCGGTACCTGGAAGCT<br>rev: CTGTCACCTCGTCGAACTCT	214
Abca1	NM_013454.3	for: CGACCATGAAAGTGACACGC<br>rev: AGCACATAGGTCAGCTCGTG	117
**Amino acid transporter**			
Snat-1	NM_001166458.1	for: AGCACAGGGCGACATTCTCATC<br>rev: ACAGGTGGAACTTTGTCTTCTTG	134
Snat-2	NM_175121.4	for: ACAAATGGGTTGTGGTA<br>rev: CCTAGATTTCTCAGCAGTGACAATG	92
**Angiogenesis**			
Flk-1	NM_001363216.1	for: GGCGGTGGTGACAGTATCTT<br>rev: GTCACTGACAGAGGCGATGA	162

Flt-4	NM_008029.3	for: GTGGCTGTGAAGATGCTGAA<br>rev: TGACACGCAAGAAGTTGGAG	199
Plgf	XM_011244016.1	for: CGTCCTGTGTCCTTCTGAGT<br>rev: CCTCTTCCTCTTCCCCTTGG	200
Vegfa	NM_001025257.3	for: CAGGCTGCTGTAACGATGAA<br>rev: GCATTCACATCTGCTGTGCT	140
Vegfb	NM_011697.3	for: AACACAGCCAATGTGAATGC<br>rev: GGAGTGGGATGGATGATGTC	157
Vegfc	NM_009506.2	for: CAAGGCTTTTGAAGGCAAAG<br>rev: TCCCCTGTCCTGGTATTGAG	159
Cd31	NM_001032378.2	for: ATGACCCAGCAACATTCACA<br>rev: CACAGAGCACCGAAGTACCA	200
hsFLT1	XM_017020485.1	for: AATCATTCCGAAGCAAGGTG<br>rev: TTTCTTCCCACAGTCCCAAC	219
**Hypoxia markers**			
HO-1	NM_010442.2	for: CACGCATATACCCGCTACCT<br>rev: CCAGAGTGTTCATTCGAGCA	175
Hif1α	NM_001313920.1	for: TCAAGTCAGCAACGTGGAAG<br>rev: TATCGAGGCTGTGTCGACTG	198
Hif2α	NM_010137.3	for: GCAAGCCTTCCAAGACACAA<br>rev: CCTGAGCTCCTGGTAGATGG	228
Phd1	NM_001357767.1	for: TGCATCAAGTGGAGGAGGAG<br>rev: GATGCTCCTGGACGGTGATA	246
Phd2	NM_001363475.1	for: AGGTGAGAAAGGTGTGAGGC<br>rev: CACAGTACAGTCCAGCAGA	228
**Trophoblast and glycogen cell marker**			
Plap	NM_007431.3	for: TCAGGGCAATGAGGTCACAT<br>rev: CCTCTGGTGGCATCTCCTTA	161
Igf-2	NM_001315489.1	for: ACCTTCCAGCCTTTTCCTGTC<br>rev: GTAGACACGTCCCTCTCGGA	357
Igf-2R	NM_010515.2	for: TATGTGAACGGCTCTGCTTG<br>rev: GAGCAAGCCTGGTCTGTTTC	200
Pcdh12	NM_017378.2	for: CTTCACCTCATCACGCTCAA<br>rev: TGCCCTCTGTCCTCTGCTAT	197
Gcm1	NM_008103.3	for: TGCTCACCTATGGCTCTCCT<br>rev: AAAATTCTGCCAAGCCCTTT	201
**Cytokine marker**			
Tnfα	NM_001278601.1	for: CGTCAGCCGATTTGCTATCT<br>rev: CGGACTCCGCAAAGTCTAAG	206

Supplementary Table S2: Antibodies used for Immunoblot and immunohistochemistry.

Primary antibody	Dilution	Host	Company	Reference number
Anti-CD31[a]	1:200 in PBS/BSA (0.5 %)	Rat	Dianova (Hamburg, Germany)	DIA-310
Anti-CD31[b]	1:5000 in 2 % milk powder	Rat	Dianova (Hamburg, Germany)	DIA-310
Anti-CX26[b]	1:350 in 2 % milk powder	Rabbit	Santa Cruz Biotechnology (Dallas, USA)	#710500
Anti-FLK1[b]	1:1000 in 5 % milk powder	Rabbit	Cell Signaling Technology (Danvers, USA)	#2479
Anti-FLT4[b]	1:200 in 0.5 % milk powder	Goat	R&D Systems (Minneapolis, USA)	AF743
Anti-GLUT1[b]	1:3000 in 2 % milk powder	Rabbit	Novus Biologicals (Centennial, USA)	NB110-39113
Anti-TNFα[b]	1:1000 in 2 % milk powder	Rabbit	Abcam (Cambridge, GB)	ab183218
Peroxidase anti-ß-actin[b]	1:20000 in TBS-T	Mouse	Sigma-Aldrich (St. Louis, USA)	A3854
Secondary antibody	Dilution	Host	Company	Reference number
Anti-goat IgG-HRP[b]	1:5000 in 0.5 % milk powder	Donkey	Invitrogen (Karlsruhe, Germany)	AB_10990162
Anti-rabbit IgG-HRP[b]	1:5000 in 0.5 % milk powder	Goat	Dako (California, USA)	p0448
Anti-rat IgG-HRP[b]	1:5000 in 0.5 % milk powder	Goat	Invitrogen (Karlsruhe, Germany)	AB_10965062
Anti-rat IgG-Biotin[a]	1:100 in 0.5 % PBS/BSA	Donkey	Invitrogen (Karlsruhe, Germany)	A18743

[a] used for Immunohistochemistry

[b] used for Immunoblot analysis.

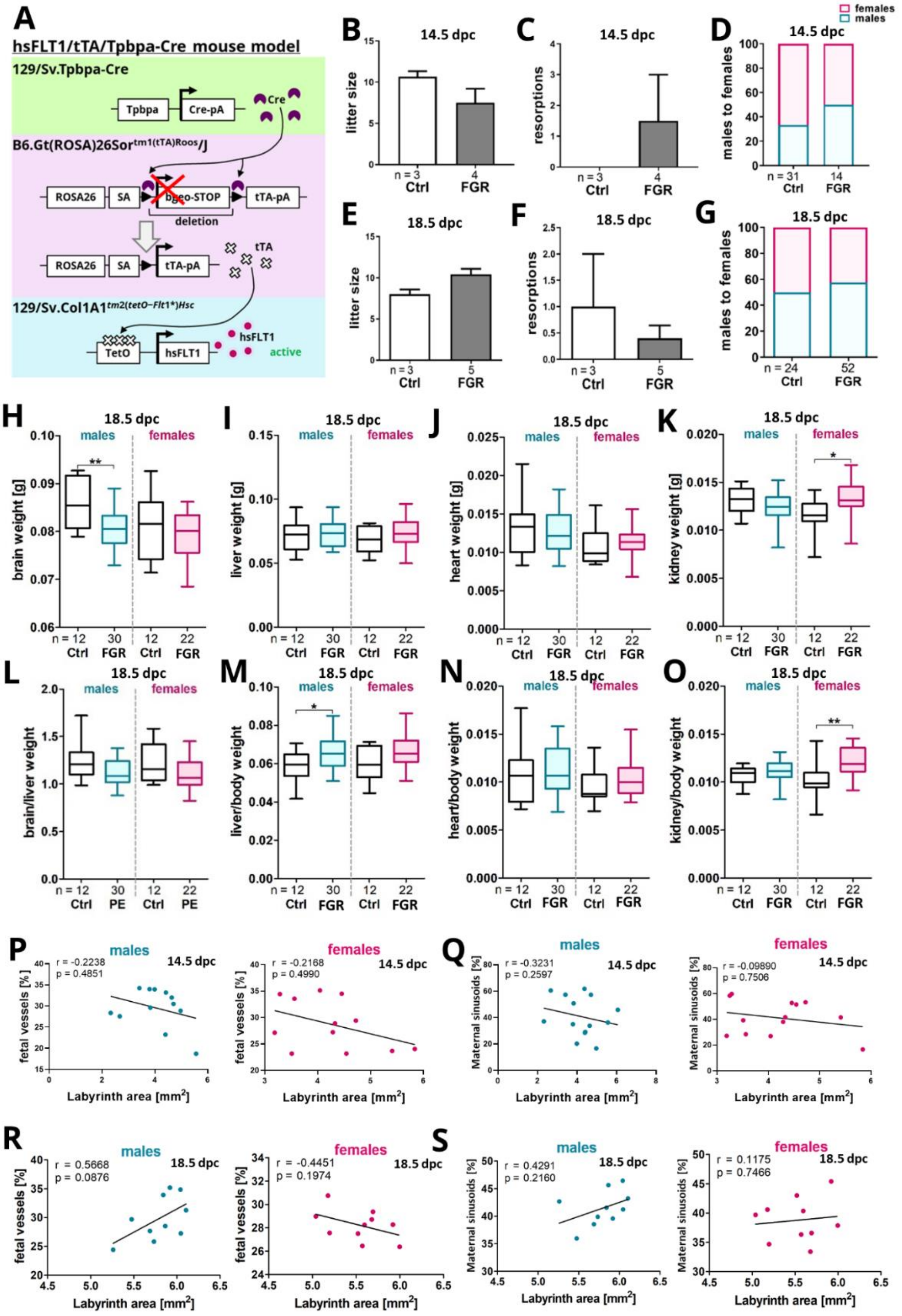

Supplementary Figure S1. Impact of placenta-specific hsFLT1 overexpression on phenotype of dams and fetuses at 14.5 and 18.5 dpc. **A**) Schematic depiction of the interaction between all three transgenes necessary to induce hsFLT1 overexpression in the hsFLT1/tTA/Tpbpa-Cre mouse model. **B/C**) At 14.5 dpc the litter size was higher in the control group (**B**), and resorptions were only observed in the FGR group (**C**). **D**) More females were born in the control group whereas the sex ratio in the FGR group was equal. **E/F**) At 18.5 dpc the litter size was higher in the FGR group (**E**), and resorptions were only observed in both groups (**F**). **G**) No change in sex ratio was observed at 18.5 dpc. **H-O**) Organ weights and organ/bodyweight ratios at 18.5 dpc: The brain weight (**H**) was significantly reduced in the FGR male group and the kidney weight was significantly increased in the female FGR group (**K**)

wherease the liver and heart weights did not change between the groups (**I, J**). The liver to body weight was significantly increased in males in the FGR group (**M**) and the kidney/body ratio was significantly increased in the females in the FGR group (**O**). The brain/liver weight and the heart/body weight did not change between the groups (**L, N**). µm **P/Q**) No Correlation of the percentage of fetal vessels (**P**) and maternal sinusoids (**Q**) with the labyrinth area was observed at 14.5 dpc **R**) At 18.5 dpc the percentage of fetal vessels correlated by trend (p=0.0876) to the labyrinth area in male FGR placentas but not in females. **S**) Whereas the percentage of maternal sinusoids did not correlate to the labyrinthine area in male and female FGR group at 18.5 dpc. Data is presented in a box plot with median, interquartile range and lower/upper extreme or in a bar graph with standard error. n=number of dams (**B/C, E/F**), fetuses (**D,G**) or fetal organs (**H-O**). * p ≤ 0.05 and ** ≤0.01 as determined by the Mann-Whitney test. Scale bar = 400 .

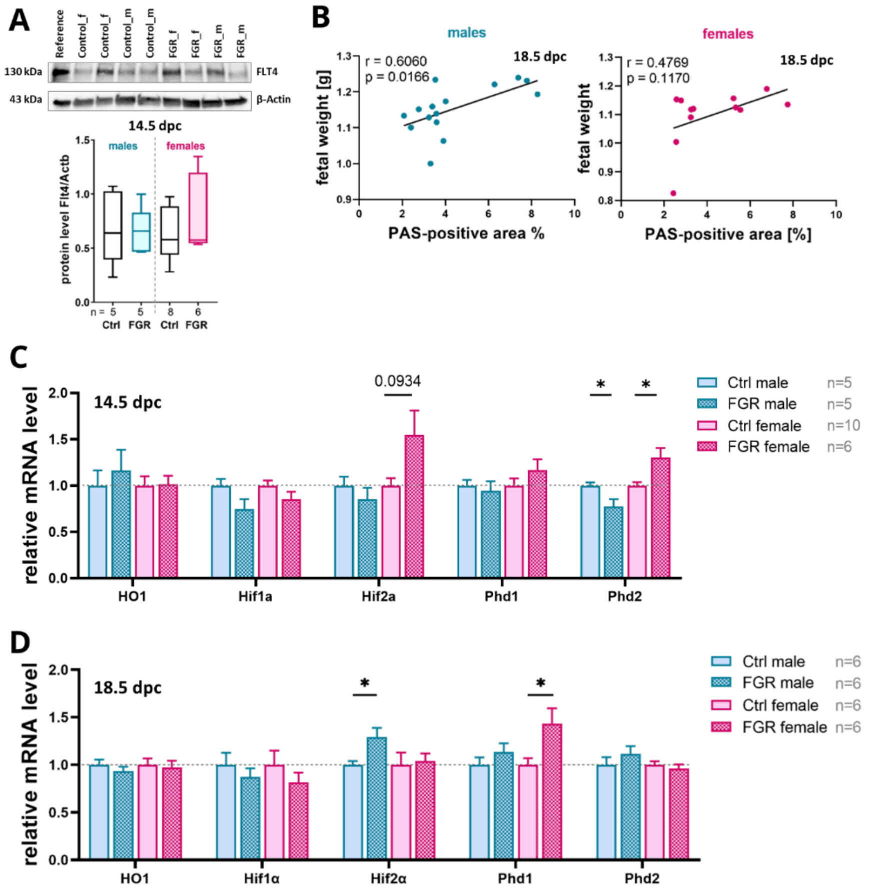

Supplementary Figure S2: Impact of placenta-specific hsFLT1 overexpression on the lymphangiogenic marker Flt4 and hypoxia-induced gene expression at 14.5 and 18.5 dpc. A) FLT4 protein level at 14.5 dpc was not changed upon placenta-specific hsFLT1 expression as shown by Western Blot analysis. **B**) The fetal weight significantly correlate to the percentage of PAS-stained area (glycogen containing cells) in the spongiotrophoblast area of male placentas but not in female placentas. **C**) Transcript levels of hypoxia/oxidative stress markers in placentas at 14.5 dpc. Only *Hif2α* was increased by trend in the female FGR groups and *Phd2* was significantly decreased in male FGR and increased in female FGR group compared to controls. **D**) At 18.5 dpc *Hif2α* mRNA was significantly increased in male FGR and *Phd1* mRNA in female FGR group. The mRNA levels were determined by qPCR and normalized to the housekeeping gene *Gapdh*. Data is presented in a box plot with median, interquartile range and lower/upper extreme or in a bar graph with standard error. n=number of placentas. * p ≤ 0.05 as determined by the Mann-Whitney test. Statistical analysis of correlation were calculated by Spearman r test.
